# Occurrence of Biosurfactant Producing *Bacillus* spp. in Diverse Habitats

**DOI:** 10.5402/2013/652340

**Published:** 2012-08-29

**Authors:** Sanket J. Joshi, Harish Suthar, Amit Kumar Yadav, Krushi Hingurao, Anuradha Nerurkar

**Affiliations:** Department of Microbiology and Biotechnology Centre, Faculty of Science, The Maharaja Sayajirao University of Baroda, Vadodara 390002, Gujarat, India

## Abstract

Diversity among biosurfactant producing *Bacillus* spp. from diverse habitats was studied among 77 isolates. Cluster analysis based on phenotypic characteristics using unweighted pair-group method with arithmetic averages (UPGMAs) method was performed. *Bacillus* isolates possessing high surface tension activity and five reference strains were subjected to amplified 16S rDNA restriction analysis (ARDRA). A correlation between the phenotypic and genotypic characterization of *Bacillus* spp. is explored. Most of the oil reservoir isolates showing high surface activity clustered with *B. licheniformis* and *B. subtilis*, the hot water spring isolates clustered in two ingroups, while the petroleum contaminated soil isolates were randomly distributed in all the three ingroups. Present work revealed that diversity exists in distribution of *Bacillus* spp. from thermal and hydrocarbon containing habitats where majority of organisms belonged to *B. licheniformis* and *B. subtilis* group. Isolate *B. licheniformis* TT42 produced biosurfactant which reduced the surface tension of water from 72 mNm^−1^ to 28 mNm^−1^, and 0.05 mNm^−1^ interfacial tension against crude oil at 80°C. This isolate clustered with *B. subtilis* and *B. licheniformis* group on the basis of ARDRA. These findings increase the possibility of exploiting the *Bacillus* spp. from different habitats and their possible use in oil recovery.

## 1. Introduction

*Bacillus* spp. are main workhorses for biotechnological applications. Their products are in the GRAS list (generally regarded as safe) of US Food and Drug Administration (USFDA) and hence are regarded harmless [1–3]. They produce a variety of products namely. extracellular enzymes, biopolymers, biosurfactants, biopesticides, and so forth from renewable resources and are ecofriendly. Biosurfactants are biologically produced surface-active compounds which are versatile process chemicals, and those from *Bacillus* spp. additionally possess the property of functionality under extreme conditions of pH, temperature, and salinity [4, 5]. These compounds have property of lowering surface and interfacial tensions of liquids. Biosurfactant production has been reported under thermophilic condition using renewable resources and by using statistically optimized medium [6, 7]. One of the potential uses of biosurfactants is in oil industry with minimum purity specification so that whole-cell broth could be used. The lipopeptide biosurfactants produced by *B. subtilis* and *B. licheniformis* are highly potent due to their surface tension reducing ability. *B. licheniformis* and *B. subtilis* have >80% identity at the nucleotide level and show extensive organizational similarity [8]. The former is facultative while the latter aerobic, both are spore forming, Gram-positive rods. Surfactin and lichenysin are two well-studied lipopeptide biosurfactants produced by *B. subtilis* and *B. licheniformis,* respectively. Similarity exists in the 25 kb, operons encoding for the surfactin and lichenysin and also in their chemical structure [9]. Accordingly their genomes are useful for comparative and evolutionary studies among species within subtilis-licheniformis group. *B. subtilis*, *B. licheniformis*, *B. cereus,* and *B. coagulans* fall in the same 16S rRNA cluster group [10].

Potent biosurfactant producing *Bacillus *species from natural habitats like oil reservoir have been reported; however, the diversity of these in various habitats has not been studied. The present investigation reveals the diversity among the biosurfactant producing* Bacillus* spp. and a distinct distribution among the species known to produce powerful biosurfactants mainly *B. licheniformis* and *B. subtilis* based on the information obtained from phenotypic characterization and amplified 16S rDNA restriction analysis (ARDRA) band patterns.

## 2. Materials and Methods

### 2.1. Sampling Sites

Diverse habitats with high salinity and/or temperatures were selected for isolation of biosurfactant producing microorganisms. Isolates available in the departmental culture collection were also included. Samples were collected from hot springs, ocean, oil wells, petrol pump, and so forth (Table 1) and were kept at 4°C until use (within 24–48 h). Temperatures of hot springs and oil wells ranged from 45°C to 65°C and from 65°C to 85°C, respectively.

### 2.2. Isolation and Initial Screening for Biosurfactant Producers

Direct isolation and enrichment technique were employed for screening and isolation of biosurfactant producers. According to Slepecky and Hemphill [10], the use of sodium chloride-peptone nitrate broth (NPNB) medium containing potassium nitrate with 5% (w/v) sodium chloride, incubation at 50°C under static condition, was used as a selective pressure for enrichment of facultatively anaerobic spore-forming bacteria. From those enriched samples, plating was carried out on NPNB plates to get the isolated colonies, and those isolated single colonies were used for screening of biosurfactant producers and also maintained on NPNB and Luria Bertani agar slants (stored at 4°C) and as glycerol stocks (stored at −20°C) for further studies. The biosurfactant producers were selected on the basis of haemolysis zone on blood agar [11].

### 2.3. Phenotypic Studies

The morphological tests included Gram-reaction, shape, motility, endospore staining, anaerobic growth, and the biochemical tests like catalase, Voges-Proskauer, nitrate reductase, gelatinase, amylase, indole production, citrate utilization, acid from glucose, gas from glucose, xylose fermentation, arabinose fermentation, mannitol fermentation, growth at 30°C, 50°C and growth in presence and absence of 5% NaCl were performed. The results of the isolates were compared to those of 19 reference* Bacillus* spp. recommended for sorting phenetic groups that is, *B. megaterium, B. circulans, B. stearothermophilus, B. licheniformis, B. subtilis, B. polymyxa, B. macerans, B. pumilus, B. coagulans, B. cereus, B. thuringiensis, B. firmus, B. alvei, B. laterosporus, B. larvae, B. popilliae, B. lentimorbus, B. brevis and, B. sphaericus* [10].

### 2.4. Bacterial Strains

The designation of the *Bacillus* spp. isolated, and the sources from which they were obtained are listed in Table 1. For ARDRA, *B. subtilis *(ATCC 6633*), B. pumilus* (NCTC 8241)*, B. cereus *(ATCC 11778)*, B. megaterium* (kindly provided by Alembic Ltd., Baroda, Gujarat, India), and* B. licheniformis *(ATCC 39307) (kindly provided by Prof. McInerney, Univ. of Oklahoma, USA) were used as standard strains.

### 2.5. Cluster Analysis

The data were analyzed using the simple matching coefficient (Sm), which considers both positive and negative results. Clustering was performed by UPGMA. The characters were coded “1” for positive and “0” for negative or absent and fed into Numerical Taxonomy System software (NTSYSpc 2.02 program). The final matrix contained 96 strains and 20 characters. The phenetic groups were sorted from the phenogram obtained by using the software.

### 2.6. DNA Extraction and PCR Amplification of 16S rRNA Gene Fragment

Genomic DNA was extracted directly from colonies on Luria Bertani agar and was used as the template for PCR [12, 13]. Universal bacterial primers-27F (5′-GAGAGTTTGATCCTGGCTCAG-3′) and 1107R (5′-GCTCGTTGCGGGACTTAACC-3′) were used for PCR amplification of 16S rRNA gene. The purity and size of each PCR product were examined by gel electrophoresis on 0.8% agarose gel in 0.5 X TBE buffer. 

### 2.7. Amplified 16S rDNA Restriction Analysis (ARDRA)

Approximately 1 *μ*g of the amplified 16S rDNA product of isolates and reference strains was taken in three separate tubes and was cleaved with 5 units of *Hae *III*, Hha *I,* and Msp *I restriction enzymes, 0.5 *μ*L of corresponding enzyme buffer was added to the assay mixture, and the final volume was adjusted to 20 *μ*L with distilled water. The reactions were carried out at 37°C for 3 h. Analysis of the reaction product was performed by agarose gel electrophoresis (2.5% w/v) in TBE buffer, containing 50 ng mL^−1^ of ethidium bromide. The gels were photographed and compared visually. The isolates and reference strains showing similar band pattern were clustered using the NTSYSpc 2.0 program after band acquisition in ALPHAEASE image aquisition software. Dendrogram based on restriction patterns of the three restriction enzymes was constructed. 

### 2.8. Surface and Interfacial Tension Measurement

Secondary screening of the biosurfactant producers was done by surface tension measurement. LB medium (50 mL in 250 mL Erlenmeyer flask) was inoculated with 2% (v/v) inoculum (OD_600_ = 1) for each isolate and incubated at 30°C; 180 rpm for 72 h. Supernatants were harvested by centrifugation, and the surface tension was measured by ring detachment method using Du-Nuoy Tensiometer (Khushboo Sci. Co., Mumbai, India), and interfacial tension (IFT) measurements against crude oil (API gravity 25) were performed in a spinning drop tensiometer (Model 510, Temco Int., USA). 

## 3. Results 

### 3.1. Phenotypic Studies and Cluster Analysis

Samples from various habitats were subjected to enrichment for facultative, sporulating, and Gram-positive rods. The isolates were checked for biosurfactant production ability on blood agar plate [11]. A total of 77 biosurfactant-producing isolates were obtained: 34 from hot water springs, 18 from oil wells, 5 from sea water, 3 from desert, one from crude oil, and 7 from petrol pump soil, indicating that biosurfactant producers were spread in all the samples selected (Table 1). Nine isolates were from departmental collection. Results for the morphological tests like Gram reaction, shape, motility, and endospore staining showed that all the isolates were Gram positive, motile, and endospore bearing short to long rods. More than 90% of the isolates were able to grow aerobically at 30°C and 50°C and showed good growth (above 0.8 OD at 600 nm) at 30°C and 50°C, in presence of 5% NaCl under aerobic conditions. All the isolates showed morphological and biochemical characteristics similar to genus *Bacillus* [10]. Based on 20 characters comprising morphological, cultural, biochemical, and physiological features (as mentioned in M&M), the isolates and the reference strains could be sorted into groups following computer analysis of the data. The isolates could be clustered in six phenetic groups, three out of them being outlier groups (I, V, and VI) with no similarity with other subgroups (II, III, and IV) (Table 2). The diversity within populations of *Bacillus* spp. obtained from petroleum contaminated soil was higher while that from the oil reservoir was the lowest. Most oil well isolates sorted in group II, hot water spring isolates within groups II and III, and petrol pump soil isolates were found to be distributed in all the three groups. Only four isolates fell in the three outgroups (Table 2).

Among the isolates belonging to the ingroups, a majority of 30 were sorted with *B. licheniformis, B. subtilis, B. polymyxa,* and *B. macerans* forming the phenetic ingroup II, 23 isolates were sorted out in ingroup III with *B. pumilus* and 20 isolates with *B. coagulans* in ingroup IV, while one isolate each in outgroups I & VI and 2 in outgroup V (Table 2). 

Among the standard *Bacillus* spp. included in the phenetic grouping, thirteen were excluded from the ingroups II, III, and IV, with three belonging to outgroup I, four to outgroup V and six to outgroup VI. The outgroup I included *B. megaterium*, *B. circulans*, and *B. stearothermophilus* with one isolate, outgroup V included along with two isolates *B. cereus, B. thuringiensis, B. firmus,* and *B. alvei *and to outgroup VI belonged* B. laterosporus, B. larvae, B. popilliae, B. lentimorbus, B. brevis,* and *B. sphaericus* (Table 2).

### 3.2. Surface Activity

All the isolates were checked for reduction in surface tension, and 24 out of 77 showed surface tension reduction below 35 mNm^−1^ (Table 3) and were selected for further studies. From them, *B. licheniformis* TT42 lowered the surface tension of water from 72 mNm^−1^ to 28 mNm^−1^ and showed IFT values 0.05 mNm^−1^ against crude oil at 80°C. IFT value for crude oil against formation water or uninoculated media was 12.4–14.0 mNm^−1^. The crude biosurfactant of *B. licheniformis* TT42 was earliar checked in the laboratory for the biosurfactant mediated microbal enhanced oil recovery (MEOR) experiments using the sand pack column models, which showed 34.6 ± 3.7% recovered residual oil [14].

### 3.3. ARDRA

**Figure 1:**
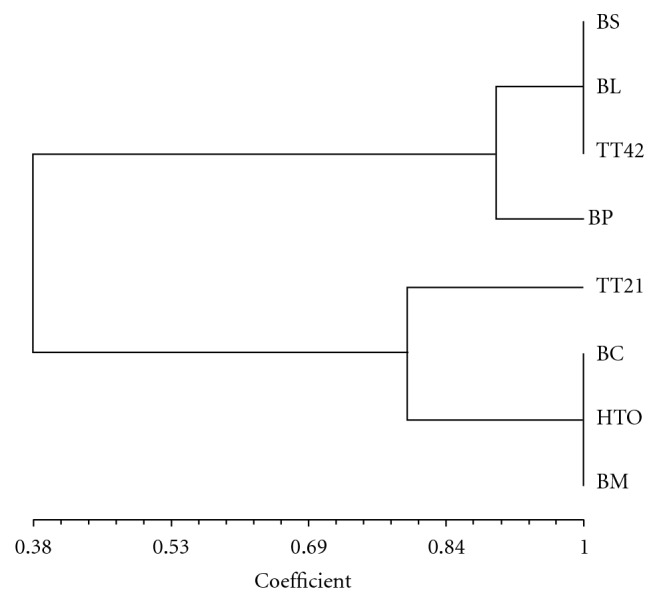
Dendrogram of genetic divergences among selected *Bacillus* isolates possessing high surface activity representing main phenetic groups and reference strains, based on restriction pattern obtained with *Hha *I*, Msp *Iand *Hae *III. BS:* B. subtilis*, BP:* B. pumilus, *BC:* B. cereus, *BM:* B. megaterium* and BL:* B. licheniformis, *Isolates TT42, TT21 and HTO.

Three biosurfactant producing isolates, TT42 and TT21 belonging to the ingroup II and HTO representing ingroup III showing surface activity between 28–35 mNm^−1^ (Table 3) and five reference strains *B. licheniformis, B. subtilis, B. cereus, B. pumilus, and B. megaterium *were subjected to ARDRA by restriction enzymes *Hae *III*, Hha *I,* and Msp *I, and dendrogram based on restriction patterns of these three restriction enzymes was constructed (Figure 1).

## 4. Discussion

Biosurfactants have the property to reduce surface and interfacial tensions of liquids. Already reported surfactants, both synthetic and natural, are capable of reducing the surface tension of water from 72 mNm^−1^ to 27 mNm^−1^ [15, 16]. Desai and Banat [5] have reported that microorganisms reducing surface tension to around 35 mNm^−1^ are probable candidates for biosurfactant production studies. In the present work, we demonstrated a reduction in surface tension to 28–35 mNm^−1^ with 24 isolates (Table 3). Bento et al. [17] demonstrated diversity of biosurfactant producing microorganisms isolated from diesel oil-contaminated soils. They isolated a total of 33 hydrocarbon-utilizing microorganisms from two soils, of which, four *Bacillus* species and one *Acinetobacter* species showed decrease in surface tension and increased emulsification activity. They demonstrated reduction in surface tension up to 41 mNm^−1^ with monoculture isolates and a defined consortium. Nazina et al. [18] have shown that the Daqing oil field, China is inhabited by aerobic saprotrophic (including hydrocarbon-oxidizing) bacteria that are able to produce oil-releasing compounds, namely surfactants and exopolysaccharides. They isolated 20 pure cultures from formation water, of which strains of *B. licheniformis* and *Rhodococcus ruber* produced efficient biosurfactant. Bodour et al., [19] have shown that the biosurfactant-producing bacteria are widely distributed in both undisturbed and contaminated soils. 

The clustering of the oil reservoir isolates with already known biosurfactant producers relates to the frequent finding of biosurfactant production in microorganisms from crude oil-containing habitats. Biosurfactants are produced by these bacteria since the environment has high hydrocarbon content. Hot water spring habitat on the other hand shows more diversity because the isolates encounter diverse nutritional environment, and absence of crude oil makes the environment less selective. On the extreme end is the petrol pump soil environment, which is found to have bacteria that are distributed in all the groups.

Phenotypic differentiation of species many times may not be straightforward; hence, results of phenetic grouping require confirmation, which was done by ARDRA. Phenetic classification using cluster and unweighted pair-group method with arithmetic averages (UPGMAs) allow clustering of like organisms and recognizes groups, which are more commonly encountered in nature. Bacteria obtained from different niches are grouped on this basis [20–23]. ARDRA, amplified ribosomal DNA restriction analysis, using different restriction enzymes and its comparison with those of well-identified species gives a fair idea of genetic grouping and aids in identification [24]. ARDRA of three biosurfactant producing isolates representing different phenetic groups and five reference strains showing high surface activity (28–35 mNm^−1^) showed polymorphism with *Hha  I*. Whereas, similar band patterns were obtained with *Hae  III* digest for the isolates and the reference strains. *Msp  I* digestion showed two patterns with standard strains. The *Msp  I* digestion pattern of *B. cereus* and isolate HTO was identical and was distinct from other standard strains. ARDRA, using *Hha *I, agreed with the results of phenetic grouping in that the *B. licheniformis* TT42 clustered with* B. licheniformis *and* B. subtilis* and isolate HTO with *B. cereus* (Figure 1). Results suggest that this oil reservoir forms a habitat for biosurfactant producers, majority belonging to *B. licheniformis* and *B. subtilis* group while diversity exists in *Bacillus* spp. from thermal and hydrocarbon containing habitats. Jennings and Tanner [25] showed that biosurfactant producers can be found in both unpolluted soils and soils polluted with hydrocarbons, and biosurfactant producing bacteria were found to constitute up to 35% of aerobic heterotrophs. There are many reports of isolation of *B. licheniformis* from oil reservoirs [18, 26]. Similar observations with petroleum-degrading bacteria were made with respect to their occurrence in special habitat [21]. 

Microbial enhanced oil recovery (MEOR) is injection of nutrients to enhance the growth of indigenous microflora in the oil well or injection of microbial products (like biosurfactants, biopolymers, acids, gases, solvents etc.), to enhance or improve the oil recovery [27, 28]. Several microorganisms produce biosurfactants, of which *B. licheniformis* among the *Bacillus* spp. produces a highly active biosurfactant lichenysin. Lichenysin is a lipopeptide biosurfactant, which can also be produced under anaerobic conditions [29]. Yakimov et al. [28] reported oil recovery efficiencies from 9.3 to 22.1% using strain *B. licheniformis *BNP29. In oil displacement experiments using *B. licheniformis* TT42, recovery of oil from sand pack column after the water flood residual oil saturation was observed [14]. A biosurfactant is of interest for petroleum industry when IFT between hydrocarbons and the culture liquid decreases at least 1000-fold [27]. Crude biosurfactant containing broth of isolate TT42 showed 250-fold, decrease in IFT from 12.5 mNm^−1^ to 0.05 mNm^−1^. An account of the lichenysin/surfactin produced by different reported *B. licheniformis* isolates reveals different properties of these strains [30]. The lichenysin of *B. licheniformis* TT42 has been found to have comparative properties to the reported strains (unpublished data). These results are promising, as the biosurfactant used in the study was crude and unpurified. 

## 5. Conclusions

The results obtained in present studies showed that isolation of biosurfactant producing microorganisms resulted in selective enrichment of spore-forming bacteria and mainly provided a better understanding of the *Bacillus* spp. predominance in this oil reservoir and other diverse habitats. The results obtained are encouraging, and further field experiments could prove the usefulness of these isolates in MEOR. Also, this knowledge can provide a rational basis for applying the MEOR effectively by doing selective enrichment of the indigenous *Bacillus* spp.

## Figures and Tables

**Table 1:** Distribution of biosurfactant producing *Bacilli* from various habitats.

No.	Source	Isolates
1	Lasundra hot water spring (Gujarat, India)	SS, N7, C, SW, N3, P
2	Tuva-Timba hot water spring (Gujarat, India)	TT11, TT12, TT13, TT14, TT15, TT21 TT22, TT23, TT24, TT31, TT32, TT33, TT34, TT36, TT41, TT42, TT44, TT46, TT51, TT52, TT61, TT62, TT47
3	Oil well (formation water; Gujarat, India)	O11, O12, O21, O22, O31, O32, O61, O62, O63, O64, O65, O6, O111, O113, O114, O121, O122, 65
4	Vrajeshwari hot water spring (Maharashtra, India)	VH1, VH2, VH3, VH4, VH5
5	Red sea (Egypt)	E1, E2, E3
6	Kutch desert soil (Gujarat, India)	KDS1, KDS2, KDS3
7	Black sea (Eastern Europe)	BS
8	Arabian Sea, Jamnagar coast (Gujarat, India)	AS-1
9	Crude oil, Institute of Reservoir Studies (Gujarat, India)	HTO
10	Petrol pump soil	P1, P3, P8, P7, P9, P4, P2
11	Departmental isolates	DI1, DI2, DI3, DI4, DI7, DI8, DI9, DI10, J

**Table 2:** Summary of the main phenetic groups and reference strains.

Phenetic group	Number of isolates	Standard *Bacillus* spp. and biosurfactant producing isolates
I (outgroup)	1	*B. megaterium*, *B. circulans*, *B. stearothermophilus*, O6
II (ingroup)	30	*B. licheniformis*, P9, TT11, TT21, TT61, TT34, TT41, TT44, P7, P, TT51, TT52, N3, *B. subtilis*, TT42, TT33, TT14, P1, TT32, TT24, TT46, SW, O21, O22, O111, O65, 65, O11, O114, *B. polymyxa*, *B. macerans*, DI2, TT12, DI4
III (ingroup)	23	*B. pumilus*, DI3, DI10, DI7, TT13, VH5, VH2, DI1, DI9, KDS3, P4, VH3, C, O63, E3, TT36, TT31, VH4, TT22, TT23, DI8, VH1, KDS2, HTO
IV (ingroup)	20	*B. coagulans*, O12, O62, O64, O61, E1, O31, O32, SS, AS-1, E2, O113, O121, BS, TT62, O122, P2, N7, P3, J, P8
V (outgroup)	2	*B. cereus*, TT47, *B. thuringiensis*, TT15, *B. firmus*, *B. alvei *
VI (outgroup)	1	*B. laterosporus*, *B. larvae*, *B. popilliae*, *B. lentimorbus*, KDS1, *B. brevis*, *L. sphaericus *

**Table 3:** Surface tension measurement of selected twenty four isolates.

No.	Isolate designation	Surface tension^*^(mNm^−1^)
1	KDS2	32 ± 1.53
2	P8	32 ± 1.15
3	P9	33 ± 0.58
4	HTO	34 ± 2.65
5	E3	32 ± 0.58
6	E2	36 ± 5.86
7	DI3	33 ± 1.73
8	VH3	34 ± 0.58
9	TT13	32 ± 1.53
10	TT33	32 ± 0.58
11	TT42	28 ± 0.58
12	TT44	31 ± 0.00
13	TT46	33 ± 1.73
14	TT47	34 ± 1.73
15	TT21	33 ± 1.53
16	O11	33 ± 1.15
17	O12	33 ± 2.08
18	O22	31 ± 1.15
19	O63	31 ± 1.00
20	O64	32 ± 1.73
21	O111	33 ± 0.58
22	O114	32 ± 1.53
23	O122	32 ± 0.58
24	O123	33 ± 0.00
26	BS^#^	50 ± 0.90
27	E1^#^	58 ± 1.00
28	AS-1^#^	55 ± 0.45
29	*B. licheniformis* ATCC 39307	30 ± 0.08
29	Control	72 ± 0.06
30	Distilled water	72 ± 0.03

^*^All the experiments were done in at least three independent experiments with SD values. Isolates were grown in LB broth (72 h; 30°C; 180 rpm).

^#^Isolates with poor surface tension reduction.

## References

[1] Food Drug Administration (1983). *21 CFR 184:1027, Federal Registration*.

[2] Schallmey M., Singh A., Ward O. P. (2004). Developments in the use of *Bacillus* species for industrial production. *Canadian Journal of Microbiology*.

[3] De Clerck E., De Vos P. (2004). Genotypic diversity among *Bacillus licheniformis* strains from various sources. *FEMS Microbiology Letters*.

[4] Banat I. M. (1995). Biosurfactants production and possible uses in microbial enhanced oil recovery and oil pollution remediation: a review. *Bioresource Technology*.

[5] Desai J. D., Banat I. M. (1997). Microbial production of surfactants and their commercial potential. *Microbiology and Molecular Biology Reviews*.

[6] Joshi S., Yadav S., Nerurkar A., Desai A. J. (2007). Statistical optimization of medium components for the production of biosurfactant by *Bacillus licheniformis* K51. *Journal of Microbiology and Biotechnology*.

[7] Joshi S., Bharucha C., Jha S., Yadav S., Nerurkar A., Desai A. J. (2008). Biosurfactant production using molasses and whey under thermophilic conditions. *Bioresource Technology*.

[8] Rey M. W., Ramaiya P., Nelson B. A. (2004). Complete genome sequence of the industrial bacterium *Bacillus licheniformis* and comparisons with closely related *Bacillus* sp.. *Genome Biology*.

[9] Ron E. Z., Rosenberg E. (2001). Natural roles of biosurfactants. *Environmental Microbiology*.

[10] Slepecky R. A., Hemphill H. E., Ballows A., Trüper T.G., Dworkin M., Harder W. (1992). The genus *Bacillus* nonmedical. *The Prokaryotes*.

[11] Carrillo P. G., Mardaraz C., Pitta-Alvarez S. I., Giulietti A. M. (1996). Isolation and selection of biosurfactant-producing bacteria. *World Journal of Microbiology and Biotechnology*.

[12] Coen D. M. (1992). *Short Protocols in Molecular Biology*.

[13] Moore D. D. (1992). *Short Protocols in Molecular Biology*.

[14] Suthar H., Hingurao K., Desai A., Nerurkar A. (2008). Evaluation of bioemulsifier mediated Microbial Enhanced Oil Recovery using sand pack column. *Journal of Microbiological Methods*.

[15] Christofi N., Ivshina I. B. (2002). Microbial surfactants and their use in field studies of soil remediation. *Journal of Applied Microbiology*.

[16] Karanth N. G. K., Deo P. G., Veenanadig N. K. (1999). Microbial production of biosurfactants and their importance. *Current Science*.

[17] Bento F. M., De Oliveira Camargo F. A., Okeke B. C., Frankenberger W. T. (2005). Diversity of biosurfactant producing microorganisms isolated from soils contaminated with diesel oil. *Microbiological Research*.

[18] Nazina T. N., Sokolova D. S., Grigoriyan A. A., Xue Y. F., Belyaev S. S., Ivanov M. V. (2003). Production of oil-releasing compounds by microorganisms from the Daqing oil field, China. *Mikrobiologiya*.

[19] Bodour A. A., Drees K. P., Maier R. M. (2003). Distribution of biosurfactant-producing bacteria in undisturbed and contaminated arid southwestern soils. *Applied and Environmental Microbiology*.

[20] Austin B., Allen D. A., Mills A. L., Colwell R. R. (1977). Numerical taxonomy of heavy metal-tolerant bacteria isolated from an estuary. *Canadian Journal of Microbiology*.

[21] Austin B., Calomiris J. J., Walker J. D., Colwell R. R. (1977). Numerical taxonomy and ecology of petroleum degrading bacteria. *Applied and Environmental Microbiology*.

[22] Mallory L. M., Austin B., Colwell R. R. (1977). Numerical taxonomy and ecology of oligotrophic bacteria isolated from the estuarine environment. *Canadian Journal of Microbiology*.

[23] Mavingui P., Laguerre G., Berge O., Heulin T. (1992). Genetic and phenotypic diversity of *Bacillus polymyxa* in soil and in the wheat rhizosphere. *Applied and Environmental Microbiology*.

[24] Caccamo D., Maugeri T. L., Gugliandolo C. (2001). Identification of thermophilic and marine bacilli from shallow thermal vents by restriction analysis of their amplified 16S rDNA. *Journal of Applied Microbiology*.

[25] Jennings E. M., Tanner R. S. Biosurfactant producing bacteria found in contaminated and uncontaminated soils.

[26] Yakimov M. M., Timmis K. N., Wray V., Fredrickson H. L. (1995). Characterization of a new lipopeptide surfactant produced by thermotolerant and halotolerant subsurface *Bacillus licheniformis* BAS50. *Applied and Environmental Microbiology*.

[27] McInerney M. J., Maudgalya S., Nagle D. P., Knapp R. (2002). Critical assessment of the use of microorganisms for oil recovery. *Recent Research in Developmental Microbiology*.

[28] Yakimov M. M., Amro M. M., Bock M. (1997). The potential of *Bacillus licheniformis* strains for in situ enhanced oil recovery. *Journal of Petroleum Science and Engineering*.

[29] Javaheri M., Jenneman G. E., McInerney M. J., Knapp R. M. (1985). Anaerobic production of a biosurfactant by *Bacillus licheniformis* JF-2. *Applied and Environmental Microbiology*.

[30] Nerurkar A. S., Sen R. (2010). Structural and molecular characteristics of Lichenysin and its relationship with surface activity. *Biosurfactants*.

